# Outcome in Advanced Ovarian Cancer following an Appropriate and Comprehensive Effort at Upfront Cytoreduction: A Twenty-Year Experience in a Single Cancer Institute

**DOI:** 10.1155/2010/214919

**Published:** 2010-07-25

**Authors:** Anne Marszalek, Séverine Alran, Suzy Scholl, Virginie Fourchotte, Corinne Plancher, Christophe Rosty, Jean Philippe Meyniel, Vincent De Margerie, Thierry Dorval, Anne De La Rochefordière, Paul Cottu, Peter Petrow, Xavier Sastre-Garrau, Rémy Jacques Salmon

**Affiliations:** ^1^Department of Surgery, Institut Curie, 25 rue d'Ulm, 75005 Paris, France; ^2^Department of Medical Oncology, Institut Curie, 25 rue d'Ulm, 75005 Paris, France; ^3^Department of Biostatistics, Institut Curie, 25 rue d'Ulm, 75005 Paris, France; ^4^Department of Pathology, Institut Curie, 25 rue d'Ulm, 75005 Paris, France; ^5^Department of Translational Research, Institut Curie, 25 rue d'Ulm, 75005 Paris, France; ^6^Department of Radiotherapy, Institut Curie, 25 rue d'Ulm, 75005 Paris, France; ^7^Department of Radiology, Institut Curie, 25 rue d'Ulm, 75005 Paris, France

## Abstract

*Objectives*. The purpose of this retrospective evaluation of advanced-stage ovarian cancer patients was to compare outcome with published findings from other centers and to discuss future options for the management of advanced ovarian carcinoma patients. *Methods*. A retrospective series of 340 patients with a mean age of 58 years (range: 17–88) treated for FIGO stage III and IV ovarian cancer between January 1985 and January 2005 was reviewed. All patients had primary cytoreductive surgery, without extensive bowel, peritoneal, or systematic lymph node resection, thereby allowing initiation of chemotherapy without delay. Chemotherapy consisted of cisplatin-based chemotherapy in combination with alkylating agents before 2000, whereas carboplatin and paclitaxel regimes were generally used after 1999-2000. Overall survival and disease-free survival were analyzed by the Kaplan-Meier method and the log-rank test. *Results*. With a mean followup of 101 months (range: 5 to 203), 280 events (recurrence or death) were observed and 245 patients (72%) had died. The mortality and morbidity related to surgery were low. The main prognostic factor for overall survival was postoperative residual disease (*P* < .0002), while the main prognostic factor for disease-free survival was histological tumor type (*P* < .0007). Multivariate analysis identified three significant risk factors: optimal surgery (RR = 2.2 for suboptimal surgery), menopausal status (RR = 1.47 for postmenopausal women), and presence of a taxane in the chemotherapy combination (RR = 0.72). *Conclusion*. These results confirm that optimal surgery defined by an appropriate and comprehensive effort at upfront cytoreduction limits morbidity related to the surgical procedure and allows initiation of chemotherapy without any negative impact on survival. The impact of neoadjuvant chemotherapy to improve resectability while lowering the morbidity of the surgical procedure is discussed.

## 1. Introduction

Epithelial ovarian cancer is diagnosed in 4,500 women per year in France and represents 3.8% of all female cancers worldwide [1]. In 2 out of 3 cases [2], it is diagnosed at an advanced stage, (stage III or IV) according to the International Federation of Gynecology and Obstetrics (FIGO) classification. It is commonly agreed that treatment should consist of primary cytoreductive surgery followed by platinum-based chemotherapy. The outcome of epithelial ovarian cancer appears highly dependent on the results achieved by primary surgery, as shown in the meta-analysis by Bristow et al. in 2002 [3]. The smaller the residual disease, the better is the prognosis. In order to achieve this minimal residual disease, surgeons have attempted more aggressive procedures with extensive resection of bowel and peritoneum in addition to the total abdominal hysterectomy, bilateral salpingo-oophorectomy (TAH-BSO), and omentectomy. Systematic pelvic and paraaortic lymph node dissections also still remain controversial. However, while the quality of surgical resection is dependent on the extent of disease and the multiplicity of peritoneal deposits, additional parameters, pertaining to tumor aggressive behavior and particularly vascularity and adhesion, may defy the best surgical skills. At Institut Curie, successive surgical teams have refrained from overly aggressive surgery, source of increased morbidity or even intraoperative, or postoperative death [4]. Our attitude over time has been that of an “appropriate and comprehensive” effort at upfront cytoreduction allowing an uncomplicated postoperative course and rapid initiation of chemotherapy in most cases. 

The disease-free and survival results of this surgical attitude followed by chemotherapy were analyzed in this retrospective study relating to this 20-year experience. Future management options, particularly chemotherapy tailored to histologic or molecular/genetic subtype, number of courses of neoadjuvant chemotherapy as well as preventive surgery in high-risk patients, and the assessment of biological factors will be discussed.

## 2. Patients and Methods

Between 1985 and 2005, 340 out of 420 patients treated for ovarian cancer at the Institut Curie with FIGO stage III or IV disease were analyzed in this retrospective study. All 340 patients had undergone primary surgery designed to achieve resection as complete as possible, that is, residual disease measuring ≤1 cm. Following initial peritoneal cytology, the operation consisted of TAH-BSO, omentectomy, possibly associated with bowel resections (appendicectomy and/or bowel resection) and multiple peritoneal biopsies. Pelvic and paraaortic lymph node dissection was performed in the presence of palpable nodes.

All histopathology slides were reviewed to assess tumor grade according to current criteria and, whenever necessary, tumors were reclassified according to their histologic type (particularly to avoid confusion between mucinous tumors and metastasis from bowel tumors) [5, 6].

Perioperative complications or death were defined as adverse events, when they occurred within 30 days of surgery according to the Memorial Sloan-Kettering Cancer surgical events grading system [7]. 

Following surgery, patients started first-line chemotherapy within a median interval of 27 days. Before 2000, chemotherapy consisted of two schedules given sequentially every 10 days (Ovaire A) for 9 injections followed by 3 weekly (Ovaire B) courses administered for 3 courses. Ovaire A consisted of Isofosfamide 1.4 g/m^2^ on days 1, 2, and 3 as well as CDDP 1 mg/kg and 5 FU 600 mg/m^2^ day, on days 1, 10, and 20. Ovaire B consisted of Isofosfamide 1.4 g/m^2^ on days 1 to 3, CDDP 75 mg/m^2^ on day 1, and 5 FU 600 mg/m^2^ on day 1. From 200 onwards, chemotherapy consisted of Carboplatin (AUC 5) and Paclitaxel 175 mg/m^2^ on day 1 every 3 weeks for 6–9 courses.

In the case of complete clinical and radiological remission following chemotherapy together with normalized CA125 values, “second look” surgery by laparotomy was considered. While earlier recommendations suggested the use of “second look” surgery to document histologic complete remission, this attitude was only routinely carried out in the context of clinical trials. In presence of residual tumor, second-line chemotherapy was initiated, the nature and duration of which depended on the patient's age, toxicity, comorbidity, and response to first-line therapy.

Statistical analysis: overall survival and disease-free survival curves were plotted according to the Kaplan-Meier method and the log-rank test. A Cox model was used to analyze the correlation between survival and the significant variables studied. A *P* value less than  .05 was considered to be significant.

## 3. Results

### 3.1. Patient Characteristics

The mean age was 58 years (range: 17–88), 53% of patients were under the age of 60, 71.3% were postmenopausal. The average parity for the entire population was two, while 20.2% of women were nulliparous.

### 3.2. Surgical Procedures

Laparotomy with a xiphoid-to-pubis incision was the preferred incision, used in 94.7% of cases. Five patients (1.5%) underwent laparoscopy only, and laparoscopy followed by laparotomy was performed in another 13 cases (3.8%). Surgical procedures are shown in Table 1. Omentectomy, TAH, and BSO were performed in 82.4%, 62.3%, and 82.1% of cases, respectively. Lymphadenectomy was neither systematic nor comprehensive; pelvic lymphadenectomy was performed in 10% of cases (unilateral: 3.8% or bilateral: 6.2%) and paraaortic lymphadenectomy was performed in 14.3% of cases. Overall, 18% of women underwent pelvic and/or paraaortic lymph node resection. Seventy seven women (22.6%) had a bowel procedure: appendicectomy (6.6%), mesenteric biopsy (9.2%), or bowel resection with immediate anastomosis (3%). The surgical findings at the end of the surgery for the entire population showed residual disease less than one centimeter in 39.8%, of which 20.3% had a complete macroscopic resection. Residual disease larger than one centimeter was present in 60.2% of patients, with diffuse peritoneal carcinomatosis in 26.7% of cases. 

The majority of patients had FIGO stage III ovarian cancer: 77.6% (comprising 22.3% cases of stage IIIc) and 22.3% had FIGO stage IV cancer. The most frequent sites of metastasis were pleura: 51.3%, liver: 10.5%, and both of these organs simultaneously: 6.6%.

The perioperative morbidity rate was 2.9% (10/340). The main complications (grade 3 to 5 perioperative morbidity) consisted of one intraoperative pneumothorax and three postoperative complications. One 78-year-old woman died at day 18 with bilateral pleural effusion and parenchymal liver metastasis, and 2 patients required redo surgery: one for postoperative bleeding and another for peritoneal abscess.

### 3.3. Histopathologic Results

Histologic examination revealed 81.5% of serous and/or papillary tumors, 7.6% of endometrioid tumors, 3.5% of undifferentiated tumors, 2.6% of mucinous tumors, and 1.2% of clear cell tumors. All tumors initially classified as “mucinous” were reviewed and reclassified. Three tumors actually corresponded to primary gastrointestinal tumors (two colonic and one pancreatic), 3 to pseudomyxoma peritonei, and 4 were finally reclassified as serous tumors.

Histologic examination of lymph node resection was negative in 21 out of 34 cases (72.4%) for pelvic nodes and in 26 out of 44 cases (53%) for paraaortic lymph nodes.

### 3.4. Chemotherapy

Nearly all patients (97.9%) received platinum-based chemotherapy after primary surgery. Seven patients opted out of chemotherapy. Median time to initiation of chemotherapy was 27 days. Patients treated before 1999 (65.2%) received a combination of platinum-based and alkylating agent chemotherapy and patients treated after 2000 (34.8%) received the Paclitaxel-Carboplatin (or CDDP) combination. 

Second-look surgery was performed in 51.5% of cases, confirming complete remission (histologically proven) in 37.7%, macroscopic residual disease in 56%, microscopic residual disease in 5.1% and isolated positive peritoneal cytology in 1.1%.

Second-line chemotherapy was prescribed in 52.3% of cases with an average of 4 cycles. Subsequent lines depended on the patient's condition and previous response.

### 3.5. Progression-Free and Overall Survivals

The mean followup was 101 months (range: 5 to 203 months). At the time of analysis, 27.9% of women treated according to this protocol were still alive, and 72% had died, due to disease progression in 96.3% of cases.

**Figure 1:**
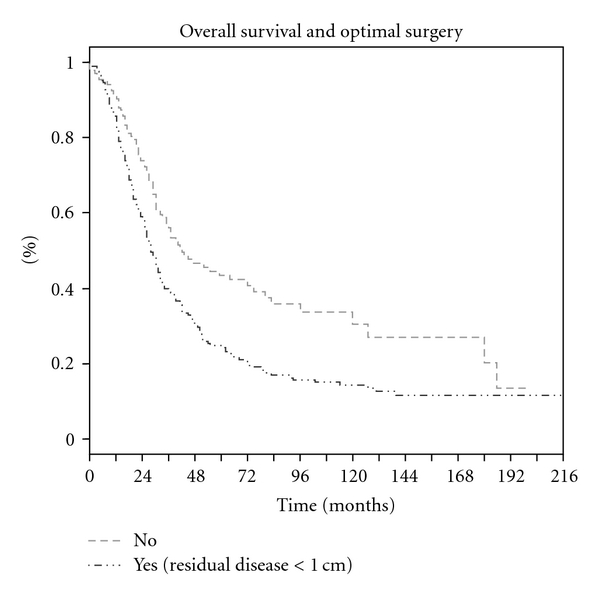
Overall survival and optimal surgery.

Median overall survival was 32 months (range: 29–38) with a five-year overall survival rate of 31.4%. The 5-year overall survival rate was 24.2% when residual disease measured more than one centimeter, 43.5% when surgery was optimal (Figure 1), and 55.4% in the absence of any residual disease. The median disease-free survival was 19 months with a five-year disease-free survival rate of 17.8%.

Univariate analysis of prognostic factors affecting five-year overall survival identified several significant factors. As shown in Table 2, the results of primary surgery, age at diagnosis, stage of disease, and results of second-look surgery were significant. Five-year progression-free survival was influenced by optimal debulking surgery, tumor histology, and type of chemotherapy.

 The Cox model was used to calculate the relative weight of the prognostic factors found to be significant for overall survival in univariate analysis. As shown in Table 3, residual disease larger than one centimeter was associated with a hazard ratio of 2.2 [1.53–3.15] (*P* = 2 · 10^−1^). Menopausal status also very significantly influenced overall survival, as the hazard ratio for postmenopausal women was 1.47 [1.10–1.96] (*P* = 7 · 10^−3^). Analysis of the type of chemotherapy showed better results for the Platinum-Paclitaxel combination compared to the platinum-alkylating agent combination, with a hazard ratio of 0.72 [0.53–0.97] (*P* = 3 · 10^−2^).

## 4. Discussion

Maximal cytoreduction was by far the most important prognostic parameter in the meta-analysis by Bristow et al. [3] which included 81 published trials and 6,885 advanced ovarian cancer patients. Depending on whether or not complete primary debulking could be achieved, the median disease-free survival was 22 months versus 14 months and the median overall survival was 52 months versus 26 months, respectively. By classifying patients in cohorts as a function of the degree of cytoreduction that could be performed, a 10% increase in the population able to receive maximal cytoreduction was associated with a 5.5% increase in median survival time. This meta-analysis therefore powerfully suggested that the primary objective of gynecologic oncology surgeons should be to attempt the most radical approach to eliminate all residual disease by the end of the primary operation. Is such a maximally aggressive surgical approach in the patient's long-term best interests? Advanced ovarian cancer patients are already frail due to their heavy tumor load, age, potential comorbidity (ASA predictive score), and nutritional status (low-preoperative albumin levels), and all of these parameters must be taken into account in the surgical decision. To achieve maximal cytoreduction in advanced ovarian cancer, some authors perform multiple visceral resections, resection of diaphragmatic tumor seedings, hepatic, gastric, duodeno-pancreatic procedures, vascular, or even urinary tract resections in addition to the standard TAH BSO and omentectomy. Although the perioperative mortality of this type of surgery is low [4], the severe morbidity rate is high, ranging from 20 to 35% according to the literature [4], while the survival gain is unclear.

The types of surgical procedures performed at Institut Curie were consistent over the years and with successive teams. Radical procedures, as described by Obermair et al. [8] and Bristow et al. [9], and particularly highly radical surgery as described by Chen et al. [10], Yildirim and Sanci [11], and Silver [12] were generally considered not to be in the patient's best interests. Particular emphasis is placed in our center on avoidance of adverse effects, such as short small bowel syndrome or permanent stoma, and the need for major vascular surgery. However, bowel resection was considered acceptable when it could be performed in a single procedure allowing either complete tumor resection or prevention of a potential risk of imminent bowel obstruction. Above all, it was considered that bowel resection must not delay initiation of chemotherapy. In the present series, the type and extent of surgical procedures beyond a “comprehensive effort at optimal cytoreductive surgery” involved bowel resections in only 3% of patients, extensive biopsies on bowel or peritoneum in 9.2%, and lymph node resection in 18%. Despite these small numbers of “aggressive procedures”, our results compare favourably with those of other studies [13–16], in which the authors performed more radical surgery with the corollary of higher intraoperative and postoperative complications (Table 4) and remain in full agreement with the data published by other centers (as reviewed by Bristow et al.). The median 5-year survival rates observed in the very restrictive group of patients who could be totally debulked (no residual disease) at the end of the primary operation was 55.3%, which compares favorably to the results reported by Silver [12] and Eisenkop et al. [13]. Of interest are the slightly better long-term results in our group of patients with postoperative residual disease >1 cm, suggesting that the better outcome of our patient population could have been potentially influenced by more rapid initiation of chemotherapy. Postoperative gastrointestinal fistulas or major fatigue as well as slow recovery of bowel function may delay initiation of chemotherapy, particularly when chemotherapy is associated with the newer antiangiogenic agents. We believe that the management of advanced ovarian cancer patients must be based on a concerted effort between surgeons, clinical oncologists, and a supportive care team. A recent retrospective study [17] has nevertheless shown that patients requiring very invasive procedures to achieve maximal cytoreduction at primary surgery have improved responses to first-line chemotherapy, and comparable outcome to those operated by so-called “standard” techniques. This study was not randomized and remains inconclusive.

The diagnostic staging role of lymph node dissection has been clearly established and has been taken into account in the FIGO classification since 1987. The frequency of lymph node invasion increases with clinical stage and ranges from 55% to 74% in stage III and 65% to 75% in stage IV patients [18–21]. While valuable for staging of early ovarian cancer, the benefit of systematic lymph node dissection remains controversial in advanced disease, in terms of curative intent. It was not performed on a regular basis as part of the primary cytoreductive surgery in our population, as we considered that the therapeutic benefit of this procedure in terms of survival has not been demonstrated in prospective trials. The recent EORTC trial [22] evaluating the impact of chemotherapy in early stage ovarian cancer emphasized the better results obtained in precisely staged patients, but understaging of the disease in advanced stages does not modify the indication for chemotherapy. Some authors consider lymph node metastases to be sites of chemoresistance; according to Wu et al. [23], lymph nodes are metastatic in 77% of cases on second-look surgery after complete primary treatment. A therapeutic benefit of lymph node dissection has been claimed by several authors based on retrospective studies. Burghardt et al. [24] reported a 5-year survival of 13% in the absence of lymph node resection compared to 53% when lymph node dissection was performed in stage III patients. In stage II, III, and IV patients, Di Re et al. [25], similarly reported a 5-year survival of 30% in the absence of lymph node resection compared to 46% following lymph node resection. However, the only randomized prospective study, by Panici et al. [26] failed to demonstrate a significant benefit in terms of overall survival, while also showing the need for more frequent transfusions and a significantly longer operating time. This milestone study, which however only accrued slowly over 12 years, randomized stage IIIb, IIIc, and stage IV patients (*n* = 427) to arms with or without systematic pelvic and paraaortic lymph node dissection. During surgery, in the case of palpable nodes in patients not randomized to systematic resection, lymph node “picking” was allowed. This study demonstrated a positive impact on disease-free survival (29.4 months versus 22.4 months) in favor of systematic lymph node dissection, but showed absolutely no effect on overall survival, with median survival times of 58.7 months and 56.3 months, respectively. In our retrospective evaluation, only 61 patients (17.9%) underwent lymph node resection of palpable nodes (>1 cm in diameter) or complete lymph node resection. Consequently, our group of patients with lymph node resections was heterogeneous and the numbers were too small to demonstrate a statistically significant difference. It should also be noted that, in the absence of complete lymph node dissection, patients with early stage ovarian cancer may have been understaged and that inclusion of these stage IIIc patients (due to node positive disease) with a better prognosis [27] than patients with peritoneal deposits, may have influenced survival data.

Patient outcome at Institut Curie was evaluated over a very long period, introducing a large number of variables over time. In this retrospective study, administration of platinum agents remained constant over the years, consisting of CDDP prior to 2000 and Carboplatin after 2000. The first-line chemotherapy protocols were fairly consistently administered to all patients with only 7 exceptions. Two thirds of the total patient population was treated by the Ovaire A-Ovaire B protocol. One third of the total population, subsequent to 2000, received a Carboplatin-Taxane regimen as first-line chemotherapy. It is noteworthy that in subsequent lines of treatment the choices of drugs varied substantially, depending on various factors, including the patient's response, toxicity, performance index and comorbidity. Interestingly, the change from pre- to post 2000 protocols did not appear to result in an enhanced progression-free survival. In fact, PFS was significantly shorter (*P* < .005) in the Carboplatin-Taxol group (median PFS of 26.7 versus 38.2 months) than in the group treated before 2000, but the more recent patient population had a small sample size with limited followup. The standard chemotherapy prior to 200 was continued for a period of 6 months, possibly delaying the onset of relapse. Seemingly in contradiction, overall survival appeared to be slightly better in the Carboplatin-Taxol group (median OS of 34.6 versus 30.3 months, (*P* < .05)), but again more recently treated patients had probably received many more subsequent lines of treatment, using newer drugs, and may have benefited from repeated surgical treatments and better supportive care. These data are consistent with several those published trials [28, 29]. 

While very few changes were made to first-line systemic therapies up until 2005 and virtually all patients received one of the two major platinum-based chemotherapy protocols, considerable progress has been made in terms of subsequent lines of treatment and other aspects of patient management such as surgical and anesthetic techniques and supportive care. More recently, specific molecular subgroups with a better response to chemotherapy, particularly to alkylating agents, have been identified, as well documented in a case-control study by the Royal Marsden hospital, describing the clinical features and outcome of patients with epithelial ovarian cancer associated with BRCA1 and BRCA2 mutations [30]. In a multivariate Cox analysis, the following 4 variables: BRCA status, stage (4 versus 2/3), age at diagnosis as well as year of diagnosis were significant parameters for outcome. Patients with the “BRCAness syndrome” appear to be associated with higher response rates, duration of response, and capability to respond to several lines of chemotherapy and merit a more systematic evaluation in the future. Prior to 2000, results on genetic testing were available in only a minority of patients in our center, and a family history of breast or ovarian cancer was also not systematically registered for all patients. 

Outcome as a function of tumor histology is another variable that must be taken into account in the future, especially as different histologic subtypes may respond to different types of chemotherapy. A systematic pathologic review of all mucinous tumors reclassified 16 of the initial 25 mucinous ovarian tumors into primary gastrointestinal tumors. Mucinous type histology may also be associated with almost twice the hazard ratio of death as compared to endometrioid type histology according to a recent report by Winter et al. [31]. Similar data have been published by other authors and molecular typing may be helpful to avoid erroneous classifications, particularly as these tumors may not be very sensitive to taxane and carboplatin regimens [32, 33]. 

In conclusion, complete surgical resection of advanced ovarian cancer is likely dependent on initial tumor volume, as complete resection is obtained more easily for less bulky tumors. However, tumor bulk is not the only parameter and biological variables may also play a role. Genomic profile analysis has been proposed as a promising tool for the future. Berchuck et al. [34] determined a genomic profile predictive of complete tumor resection, suggesting that completeness of resection may depend on the inherent biological aggressiveness (or indolence) of the tumor. Tumor stromal and proangiogenic properties as well as its immunological characteristics and inflammatory cytokine profiles may all influence tumor adhesive and invasive properties and thereby influence the ease of tumor resection. 

In the near future, primary cytoreductive surgery remains the primary option of treatment for advanced ovarian cancer patients, but we feel that highly mutilating surgery may be foregone by an upfront systemic therapy which should ideally be tailored to the tumor molecular characteristics. The number of courses of systemic therapy remains another subject of debate, but possibly the most beneficial approach for the patient would be an individualized duration of treatment until best response as evaluated by serum markers.

## Figures and Tables

**Table 1:** Surgical procedures performed during primary cytoreductive surgery.

Surgical procedures	Number of patients	Percentage
Hysterectomy:		
total	152/340	44.7%
subtotal	61/340	17.9%

*Salpingo-oophorectomy* (unilateral or bilateral)	279/340	82%

*Omentectomy*	276/335 (5 MD)	82.4%

*Bowel procedures:*		
Appendicectomy	22/335	6.6%
Bowel or peritoneal biopsies	31/335	9.2%
Resection	10/335	3%
Other	4/335	1.2%

*Lymph node resection (picking)*		
Pelvic	34/340	10%
Paraaortic	44/308 (32 MD)	14.3%
Pelvic and/or paraaortic	61/340	18%

MD = missing data.

**Table 2:** Predictive factors of survival.

Predictive factors	Number of patients	Overall survival		Disease-free survival	
		5-year survival (%)	*P*	5-year survival (%)	*P*
*Age*					
<60 years	180	36.1	*P* = .002	27.7	*P* = .68
≥60 years	160	26.1		26.9	

*Menopausal status*					
No	95	43.7	*P* = .001	31.5	*P* = .128
Yes	237	36.4		25.4	

*Residual disease after surgery*					
<1 cm (without residual disease)	135 (69)	43.5 (55.4)	*P* = .0002	27.6 (40.6)	*P* = .58
≥1 cm	204	24.2		26.7	

*Lymph node dissection (picking)*					
No	279	29	*P* = .02	24.8	*P* = .01
Yes	61	42.5		37.8	

*FIGO stage*					
III	264	34.6	*P* = .002	25.7	*P* = .15
IV	76	19.1		33.6	

*Histology*					
Serous	277	31.2		26.3	
Mucinous	9	22.2	*P* = .005	48.6	*P* = .0007
Endometrioid	26	45.0		42.9	
Clear cell	4	25.0		25.0	
Undifferentiated	12	38.9		—	
Other	12	8.3		—	

*Grade (2 classes)*					
1	13	58.6	*P* = .005	60.0	*P* = .05
2 and 3	140	24.8		16.0	

*Chemotherapy*					
Platinum and alkylating agents	217	30.3	*P* = .107	38.2	*P* = .02
Platinum and Paclitaxel	116	34.6		26.7	

NS = not significant.

**Table 3:** Multivariate analysis of overall survival (Cox model).

*N* = 324	Hazard ratio	*P*
*Optimal surgery*		
Yes	1	*P* = 7 · 10^−3^
No	2.2 [1.53–3.15]	

*Menopausal status*		
No	1	*P* = 2 · 10^−4^
Yes	1.47 [1.10–1.96]	

*Chemotherapy *		
Platinum/alkylating agents	1	*P* = 3 · 10^−2^
Platinum/taxanes	0.72 [0.53–0.97]	

**Table 4:** Comparison with other studies.

Author, year	*n*	% stage IV	Optimal surgery		5-year survival	
			Residual disease	%	Residual disease	%
Eisenkop et al., 1998 [13]	163	17%	0 cm	98.8%	0	52%
					>0	29%

Scarabelli et al., 2000 [14]	66	11%	<2 cm	100%	0	42.2%
					>0-1	21.3%

Chi et al., 2001 [15]	282	23%	<1 cm	26%	0-1	50%
					>1-2	28%
					>2	21%

Eisenkop, et al., 2003 [16]	408	0%	<1 cm	96%	0-1	52%
					>1-2	30%
					>2	0%

Present study	340	22.75%	<1 cm	40%	0	55.4%
					>0-1	43.5%
					>1	24.2%
